# 5,7-dimethoxyflavone inhibits hepatocellular carcinoma progression via increasing intestinal *Akkermansia muciniphila* and hepatic CD8^+^ T cell infiltration

**DOI:** 10.1186/s13020-025-01233-8

**Published:** 2025-10-08

**Authors:** Weicong Chen, Changshun Liu, Xiao Li, Xuemei Yang, Yang Liu, Mengchen Qin, Wentao Jiang, Yiqin Wang, Haitao Sun, Guohuan Li, Bin Wen, Songqi He

**Affiliations:** 1 Department of Hepatology, Zhuhai Hospital Affiliated to Southern Medical University (Zhuhai Hospital of Integrated Traditional Chinese and Western Medicine), Zhuhai, 519020 China; 2 School of Traditional Chinese Medicine, Southern Medical University, Guangzhou, 510515 China; 3 Department of Pathology, Guangdong Provincial Hospital of Traditional Chinese Medicine, Guangzhou University of Chinese Medicine, Guangzhou, 510120 China; 4 Department of Traditional Chinese Medicine, The Air Force Hospital of Southern Theatre Command of People’s Liberation Army, Guangzhou, 510602 China

**Keywords:** Hepatocellular carcinoma, 5,7-Dimethoxyflavone, Gut microbiota, Antitumor immunity, NF-κB/CCL2 pathway

## Abstract

**Background:**

Hepatocellular carcinoma (HCC) mainly develops in cases of fibrosis and cirrhosis and is accompanied by intestinal flora disorder. HCC also affects CD8^+^ T cell immune function. 5,7-Dimethoxyflavone (DMF), an active flavonoid with anti-tumor effect, is found in *Kaempferia parviflora*. However, whether DMF can treat HCC remains unclear. This study aims to investigate the effect of DMF on HCC and to explore its possible mechanism, focusing on the gut microbiota regulation and the effect of CD8^+^ T cells in a murine model.

**Methods:**

The HCC mouse model was induced with diethylnitrosamine/carbon tetrachloride and orally administered DMF. DMF effects on HCC progression were assessed using hematoxylin and eosin staining, immunohistochemistry, and serum biochemical marker levels. The causal relationship between gut microbes and HCC was explored using 16S rRNA genome-derived taxonomic profiling, microbial transplantation, fecal high-throughput targeted metabolomics, and untargeted serum metabolomic analyses. Transcriptome analysis, molecular docking, quantitative real-time polymerase chain reaction, and Western blot were applied to study the genes targeted by DMF. CD8^+^ T cell infiltration and tumor-killing factors were studied using flow cytometry and immunofluorescence staining.

**Results:**

DMF reduced the number of tumors, the largest tumor size, and the liver-to-body ratio while also improving liver function. An antibiotic cocktail lowered the anti-tumor effect of DMF, indicating that DMF inhibition of HCC is partially dependent on the gut microbiota. DMF considerably upregulates *Akkermansia muciniphila* during chemical hepatocarcinogenesis in mice. DMF-upregulated *A. muciniphila* leading to intestinal barrier repair, which inhibited HCC progression by enhancing antioxidant capacity through glutathione regulation and 11,12-DIHETrE down-regulation. An untargeted serum metabolomic analysis showed that there existed additional mechanisms underlying DMF anti-tumor effect following its absorption into the bloodstream. DMF enhances the infiltration effect of CD8^+^ T cells and upregulates interferon-gamma expression in HCC tissue. Overall, 822 genes, including chemokine (C–C motif) ligand 2 (CCL2), were significantly downregulated by DMF treatment in HCC cells. Notably, DMF binds strongly with nuclear factor kappa-B (NF-κB) and inhibits NF-κB p65 phosphorylation, sequentially suppressing the expression of downstream protein CCL2, which mediate the crosstalk between tumor cells and CD8^+^ T cells.

**Conclusion:**

DMF improves *A. muciniphila*-mediated intestinal barrier repair and inhibits the NF-κB/CCL2 pathway in HCC cells, enhancing the immunity of CD8^+^ T cells in the liver. Hence, it may serve as a potential candidate for HCC treatment.

**Graphical Abstract:**

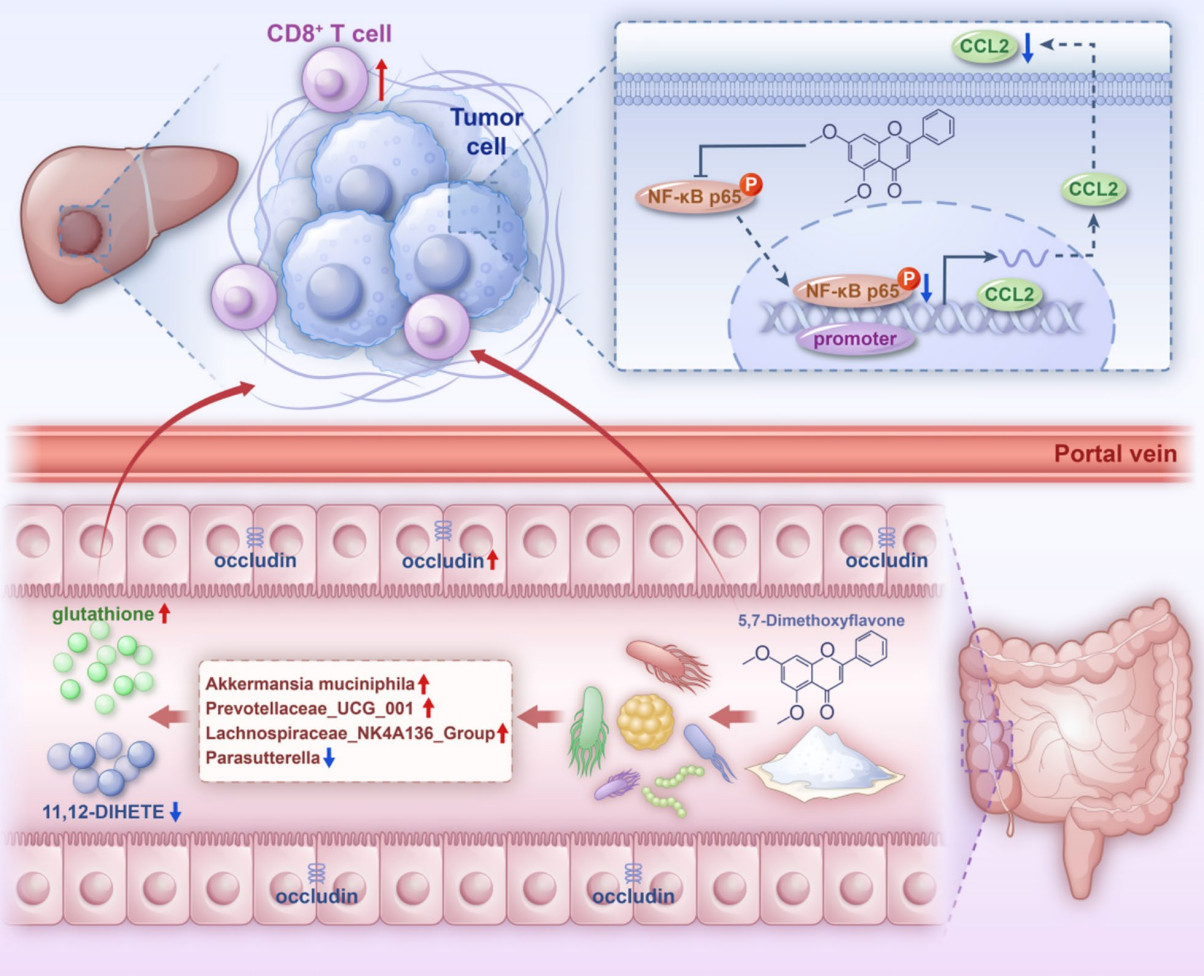

**Supplementary Information:**

The online version contains supplementary material available at 10.1186/s13020-025-01233-8.

## Highlights

5,7-Dimethoxyflavone (DMF) inhibits hepatocellular carcinoma (HCC) progression in a murine model.

DMF increases *A. muciniphila*-mediated intestinal barrier repair, preventing HCC.

DMF inhibits HCC progression by enhancing CD8^+^ T cell-mediated anti-tumor immunity.

**Supplementary Information:**

The online version contains supplementary material available at 10.1186/s13020-025-01233-8.

## Introduction

Liver cancer is the sixth most commonly diagnosed malignant tumor and the third leading cause of cancer-related mortalities globally [1]. Hepatocellular carcinoma (HCC) is the pathological type of primary liver cancer, and it accounts for over 80% of cases [2]. The infection of hepatitis viruses(hepatitis B or C virus), the metabolic dysfunction, alcohol intake, and immune dysfunction are the leading causes of HCC. Notably, there are various treatment methods of HCC include hepatectomy, liver transplantation, ablation therapy, transarterial chemoembolization, radiotherapy, systemic anti-tumor therapy, and other means. Among these, immune checkpoint inhibitors (ICIs), exerting an anti-HCC effect by enhancing the immune-attacking ability of effector CD8^+^ T cells in the tumor microenvironment, represents a breakthrough in the field of HCC treatment [3]. However, a low response rate to ICIs presents challenges in HCC immunotherapy [4].

HCC is a classic inflammation-promoting cancer that mainly develops in cases of fibrosis and cirrhosis. The gut and liver are intricately connected through the portal vein and biliary circulation. Reportedly, HCC development is mainly accompanied by gut microbiome disorders and intestinal barrier dysfunction [5]. Under pathological conditions, diverse factors disrupt the balance of the intestinal tract, resulting in intestinal flora imbalance and damage to the intestinal mucosal barrier. Intestinal bacteria and their metabolites trigger and exacerbate inflammation in the liver and intestines through the gut-liver axis, ultimately contributing to HCC development [6].

Nuclear factor kappa-B (NF-κB) signaling is crucial in HCC because it regulates multiple aspects of tumor biology, including promoting tumor cell proliferation, apoptosis inhibition, and immunosuppression modulation [7]. Chemokine (C–C motif) ligand 2 (CCL2) and NF-κB downstream target factors, can recruit tumor-associated macrophages, myeloid-derived suppressor cells, and T helper 2 cells, creating an immunosuppressive microenvironment that enables tumor progression [8]. Low expression of CCL2 increases the number of CD8^+^ T cells in hepatocellular carcinoma tissues, showing a good prognosis in patients with HCC [9].

*Kaempferia parviflora*, a species belonging to the genus Kaempferia of the family Zingiberaceae, has been extensively used as a food supplement in traditional practices in Southeast Asia and Thailand with a history of over 1000 years [10]. *K. parviflora* is reported for the treatment of various ailments, general pains, gout, hypertension, ulcer, fatigue, metabolic disorder, cognitive impairments, infections, and tumors [11]. Flavonoids, a class of polyphenolic compounds, are widely present in medicinal plants, possessing various biological activities owing to their potential antioxidant, anti-inflammatory, antibacterial, antiviral, neuroprotective, and anti-tumor effects [11]. Notably, 5,7-Dimethoxyflavone (DMF) is a natural dietary flavonoid found in *K. parviflora* [12]. *K. parviflora* extract inhibits the growth of human bile duct cancer cells and can be used to treat cholangiocarcinoma [13]. DMF could induce apoptosis in endometriosis cell lines [14], attenuate obesity by inhibiting adipogenesis [15], and increase the blood concentrations of drugs by decreasing cytochrome P450 expression in the liver [16]. However, its role and mechanisms in HCC remain unclear.

Flavonoids can disrupt the lipid bilayer of the cell membrane of harmful bacteria or affect the permeability of their cell membranes to inhibit the growth of harmful bacteria in the intestine. These compounds also provide metabolic substrates for the gut microbiota, enabling the growth of beneficial bacteria and thus optimizing the gut microbiota structure [17]. We speculated that DMF could also alleviate gut microbiota dysbiosis in HCC since it belongs to the class of flavonoids. Therefore, we explored the mechanisms of DMF in regulating gut microbes and enhancing CD8^+^ T cell immune function using 16S rRNA genome-derived taxonomic profiling, microbial transplantation, metabolomic analyses in a murine HCC model induced with diethylnitrosamine (DEN)/carbon tetrachloride (CCl_4_). Transcriptome analysis and molecular docking were used to study the genes targeted by DMF. Further, CD8^+^ T cell infiltration and tumor-killing factors were studied using flow cytometry and immunofluorescence staining.

## Materials and methods

### Reagents and chemicals

We purchased DEN, CCl_4_, and mineral oil from Sigma-Aldrich (#N0756, $1601168, #8042-47-5, St. Louis, MO, U.S.A), and ampicillin, metronidazole, neomycin, and vancomycin from Macklin (#69-53-4, #443-48-1, #1405-10-3, #1404-90-6, Shanghai, China). The other reagents used in this study were DMF (purity ≥ 99%, HY-N5011, MedChemExpress LLC, NJ, USA), Immunohistochemistry (IHC) kit (#GK5007, Gene Tech, Shanghai, China), Alpha-fetoprotein (AFP) DuoSet enzyme-linked immunosorbent assay (ELISA) kit (DY5369-05, R&D Systems, MN, USA), mouse CCL2 ELISA kit (CSB-E07430m, CUSABIO, Wuhan, China) and PowerFecal DNA extraction kit (#51804, Qiagen, Hilden, Germany). The antibodies used in this study are shown in Supplementary Table 1.

### *Akkermansia* muciniphila culture

*A. muciniphila* (strain:ATCC BAA-835) was obtained from the Guangdong Microbial Culture Collection Center (NO.1.1346) and cultured anaerobically in a liquid medium at 37 °C [18]. The liquid medium contained the brain–heart infusion broth (R060270, Thermo Fisher Scientifc, Waltham, MA, USA), 0.5% porcine mucin (S12065, Yuanye Biotechlogy, Shanghai, China) and 0.05% L-cysteine (#52-90-4, Sigma-Aldrich, Louis, MO, U.S.A).

*A. muciniphila* was initially activated anaerobically for 24 h, and subsequently diluted to an initial optical density (OD600) of about 0.1 in fresh BHI-mucin medium for growth curve measurement. Experimental groups contained DMF at a final concentration of 50 µM, while controls lacked supplementation. Bacterial growth was anaerobically monitored at 37 °C by measuring OD600 every 6 h for 72 h. We conducted each experiment in triplicate.

### Animal experiment

Both sexes of C57BL/6 J mice, aged 6–8 weeks, were obtained from the Experimental Animal Center of the Southern Medical University (no: SCXK (Yue) 2021-0041). The mice were allowed to mate freely to generate offspring for subsequent experiments. The HCC model used in this study was based on a previous study [19]. Briefly, male mice were administered a single intraperitoneal (i.p.) injection of DEN (25 mg kg^–1^) on postnatal day 14. DEN-initiated mice were administered an intraperitoneal injection of 0.5 mL kg^–1^ CCl_4_ 4 weeks later, diluted 1:10 in sterile mineral oil, for 12 consecutive weeks, as a promoting agent to induce carcinogenesis. Control mice were administered phosphate buffered saline (PBS) for 2 weeks, followed by 12 weekly injections of PBS.

The mice were randomly assigned to four groups after 2 weeks: Control, HCC, low-dose (40 mg·kg^–1^) DMF (DMF_L), and high-dose (80 mg kg^–1^) DMF (DMF_H) groups; *n* = 6 for each group. This dose has been used in previous animal studies in which mice were used [15]. Mice in the HCC, DMF_L, and DMF_H groups were treated with the DEN-CCl_4_ regimen, whereas the control group received PBS. The mice received different doses of DMF using a gavage to explore the anti-tumor effect of DMF, starting at the beginning of week 18 and continuing until the mice were sacrificed.

Mice were randomly assigned to three groups: control, HCC, and *A. muciniphila* group, *n* = 6 for each group, to explore the anti-tumor effect of *A. muciniphila*. The mice received similar treatment to that used in the first modeling method when they were between 2 and 18 weeks of age. At week 18, the *A. muciniphila* group mice were orally administered 200 μL of the *A. muciniphila* microbiota suspension, dissolved in oxygen-free PBS with a final concentration of 1 × 10^9^ CFU mL^–1^, twice a week for 6 weeks. The mice in the control and HCC groups received the same volume of vehicles.

Mice were randomly assigned to four groups: Control, HCC, DMF_H, and high-dose DMF with antibiotic cocktail (DMF_H + Abx) group, *n* = 6 for each group, to explore whether the anti-tumor effectiveness of DMF is independent of gut microbiota. The mice received a similar treatment to that used in the first modeling method. Additionally, gut sterilization was conducted in the DMF_H + Abx group using a cocktail of four non-absorbable and broad-spectrum antibiotics (ampicillin, neomycin, and metronidazole, 1 g L^–1^ each; and vancomycin, 0.5 g L^–1^) in drinking water [20], at the start of week 18 and continued until sacrifice.

All mice were sacrificed at 24 weeks of age after a 12 h fast. Blood, fecal, liver, and tumor samples were obtained for subsequent experiments.

### MR imaging

A Bruker 70/16 PharmaScan animal MRI system at 7 T (Bruker, MA, USA) was applied to image mouse liver as previously described [19]. Initially, mice were anesthetized with a mixture of 98% oxygen and 2% isoflurane. Subsequently, inhale isoflurane gas was continued at a flow rate of 2 L/min to maintain a deep anesthesia state. MRI scanning was performed. The T_2_-weighted imaging parameters were as follows: repetition time 3000 ms, echo time 30 ms, acquisition matrix = 320 × 320, 30 slices, and 0.8 mm slice thickness. The liver T2-weighted images were analyzed using Radiant DICOM Viewer software.

### Serum biochemical analysis and ELISA detection of AFP and CCL2

Mouse blood was obtained and centrifuged for 15 min at 3000 rpm. The liver function biomarkers, including aminotransferase (ALT) and aspartate aminotransferase (AST), were estimated using a Mindray automatic biochemical analyzer (BS-330E). Serum AFP and CCL2 levels were determined using a commercially available ELISA kit following the product manual.

### Histopathological, immunohistochemical and immunofluorescence staining

Liver tissue was removed and fixed with 4% formalin for 24 h. Subsequently, the tissue was embedded in paraffin, and tissue sections of 4 μm were prepared. The samples were dewaxed with xylene, dehydrated using different concentrations of alcohol, and stained with hematoxylin and eosin.

For the immunohistochemical staining, antigen was retrieved from the dewaxed and dehydrated samples. 0.3% hydrogen peroxide was used to block the endogenous peroxidase activity. The sections were incubated with primary antibody, AFP, Ki67, and occludin (both at 1:100 dilution) overnight at 4 °C. Immunostaining was conducted using an IHC kit. Finally, the sections were stained with hematoxylin. A light microscope was used to acquire image sections for subsequent analysis.

Immunofluorescence staining was performed. OCT-embedded tissue was sliced into 10-μm-thick sections. After blocking, the sections were incubated with diluted primary antibodies against CD8 (1:100) overnight at 4 °C. We washed the samples and incubated them with AlexaFluor^®^ 594 conjugated goat anti-rat IgG secondary antibody and counterstained with DAPI. Protein expression was analyzed using a Nikon microscope.

### 16S rRNA amplicon sequencing analysis

Extraction of bacterial DNA from fecal samples was conducted using the PowerFecal DNA extraction kit following the product manual. PCR amplification was conducted using universal 16S primers that targeted the V3–V4 region of the bacterial 16 s ribosomal RNA gene (forward primer 5′-TACGGTTACCTTGTTACGACTT-3′ and reverse primer 5′-AGAGTTTGATCCTGGCTCAG-3′) [21], followed by purification with gel extraction. The final DNA concentration was measured using a NanoDrop 2000 UV spectrophotometer. Thereafter, an amplification sequencing library pool was designed using the Illumina MiSeq platform by the BioProfile Biotechnology Company. Raw sequence reads were filtered by removing the last 20 bases of read R1 and the last 40 bases of read R2. The reads were processed using QIIME to select operational taxonomic units having a 97% sequence similarity [22]. The operational taxonomic units (OTUs) with a frequency < 0.05% of the total recorded frequency were removed. Finally, the OTUs were normalized, and the data were rarefied by random sampling without replacement at the lowest common sequencing depth.

### The untargeted serum metabolomics analysis

We mixed 100 µL serum with 400 µL of cold methanol: acetonitrile (v/v, 1:1) via vortexing, followed by ice-bath ultrasonic treatment for 1 h. This mixture was incubated at − 20 °C for 1 h, followed by centrifugation at 14,000 × g for 20 min at 4 °C. The supernatant was subsequently collected and dried using a Termovap sample concentrator. We resuspended the pellet in methanol for subsequent analysis.

Metabolomic profiling was conducted by the BioProfile Biotechnology Company (Shanghai, China) on a Shimadzu Nexera X2 LC-30AD ultra-high-performance liquid chromatography (UHPLC) (Shimadzu, Japan) coupled with Q-Exactive Plus (Thermo Fisher Scientifc). The liquid chromatography (LC) separation details and mass spectrometer (MS) data acquisition were analyzed as previously reported [23].

### Quantitative medical targeted metabolomics1000 (QMT1000)

Metabolite extraction of each sample was weighed and recorded. Pre-cooled 80% methanol–water was added at a weight-to-volume ratio of 1:20. Subsequently, three steel beads were added, and low-temperature homogenization and fragmentation were conducted in a tissue disruptor. Mixed thoroughly using vortexing, we conducted sonication in an ice bath for 20 min and let it stand at – 20 °C for 1 h. Subsequently, the mixture was centrifuged, and the supernatant was collected and evaporated it to dryness in a high-speed vacuum concentrating centrifuge. Redissolved the sample in pre-cooled QMT1000 working solution (with an internal standard of 50% methanol, with a water ratio of 1:9) and centrifuged it.

We collected the supernatant for mass spectrometry injection analysis [24], which was conducted using BioProfile Biotechnology. The samples were placed in a 4 °C autosampler and separated using a Shimadzu Nexera X2 LC-30AD UHPLC coupled with an HSST3 chromatographic column during the entire analysis process.

### Cell culture

The mouse HCC cell line Hepa1-6 was obtained from the National Collection of Authenticated Cell Cultures and cultured in Dulbecco’s modified Eagle’s medium (DMEM) supplemented with 10% fetal bovine serum (FBS) and 1% penicillin–streptomycin at 37 °C in a humidified atmosphere containing 5% CO_2_. After 24 h, the culture medium was refreshed with the medium containing low-dose DMF (25 µM, defined as DMF_L) and high-dose DMF (50 µM, DMF_H). The cells were collected for further analysis 24 h after incubation. Hepa1-6 cells were treated with NF-κB agonist Compound 32 (10 µM, E1351, Selleck, Shanghai, China) or inhibitor Bay 11-7082 (1 µM, S2913, Selleck) to investigate the pharmacological effect of DMF on the NF-κB pathway. High-dose DMF or combined application of Compound 32 and DMF were also used.

### CCK8 assay

5 × 10^3^ Hepa1-6 cells were seeded into the 96-well cell plate, and incubated with 4% CO_2_ at 37 °C for 24 h. After DMF (3.125, 6.25, 12.5, 25, 50, 100, 200, 400 µM) treatment for 24 h, cell viability was measured using cell counting kit-8 (CCK8) assay. After the treatments of DMF, 20 µL CCK8 was added to each well, incubated for 1 h at 37 °C and measured at 570 nm using a microplate reader (Thermo, MA, USA) for calculating cell viability.

### Transcriptome analysis

Transcriptome analysis was performed by Beijing Genomics Institute (Shenzhen, China). Briefly, total RNA was extracted from the cultured cells using RNA Extraction Kit (AG21017, Hunan, China) according to instructions, and total RNA was qualified. Subsequently, mRNA library construction was performed. Next, purified mRNA was fragmented into small fragments to generate cDNA via reverse transcription. The cDNA was amplified, and the products were purified, which were used to construct the final library and analyzed using the BGIseq 5000 platform. Sequencing data were filtered with SOAPnuke and the clean reads were aligned with the gene set using Bowtie2 [25]. KEGG (https://www.kegg.jp/) and GO (http://www.geneontology.org/) enrichment analyses of differential gene expression were performed based on hypergeometric test.

### Quantitative real-time PCR (RT-PCR)

Total RNA extraction was performed utilizing SteadyPure Universal RNA Extraction Kit (AG21017, Accurate Biotechnology, Hunan, China). According to the manufacturer’s instructions, complementary DNA was synthesized using an Evo M-MLV reverse transcription Kit (AG11711). LightCycler 96 (Roche, Basel, Switzerland) was applied for fluorescent RT-PCR amplification using SYBR^®^ Green Premixed qPCR Kit (AG11759). The RT-PCR protocol was as follows: 95 °C for 30 s, followed by 40 amplification cycles of 95 °C for 10 s, then annealing at 60 °C for 30 s, and finally at 95 °C for 15 s. The changes in gene expression levels were analyzed using 2^−△△Ct^. Primer details are listed in Supplementary Table 2.

### Western blot (WB)

Cell lysates were loaded onto a 10% SDS–PAGE gel and then the proteins were transferred to nitrocellulose membrane (Millipore, MA, USA). Primary antibodies against CCL2, NF-κB p65, p-NF-κB p65, extracellular regulated protein kinases 1/2 (ERK1/2), p-ERK1/2, signal transducer and activator of transcription 3 (STAT3), p-STAT3, and GAPDH (all at a dilution of 1:1000) were incubated with the membranes. Subsequently, the membranes were incubated with secondary antibodies for 1 h. Finally, the immunoblots were analyzed using Bio-Rad chemiluminescent imaging system. ImageJ was used to measure the band intensities.

### Flow cytometry

HCC tissues were resected, minced into small pieces, and digested enzymatically at 37 °C with 40 mL DMEM containing 1 mg·mL^−1^ type IV collagenase (C5138, Sigma), 0.1 mg·mL^−1^ hyaluronidase (H1115000, Sigma), and 0.01 mg·mL^−1^ DNase I (#10104159001, Roche) [26, 27]. After 45 min, the digestion was discontinued using DMEM containing 2% FBS, passed through 70-μm cell strainers, followed by centrifugation at 50 × g for 5 min to separate hepatocytes and non-parenchymal cells. The supernatant was pelleted by centrifuging at 400 × g for 5 min before it was washed with buffer (DMEM containing 1% FBS). Red blood cells were lysed, followed by washing twice and centrifugation. The cells were transferred into a 24-well plate (10^6^ cells·mL^−1^), and mixed reagent, Leukocyte Activation Cocktail, with BD GolgiPlug^™^ (BD Pharmingen), containing phorbol 12-myristate-13-acetate, ionomycin, and Brefeldin A at a volume of 2 μL·mL^−1^ was added.

After incubation for 6 h, the stimulated cells were washed and centrifuged at a speed of 350 g for 5 min. This process was repeated. The pellet was resuspended in a buffer for flow cytometric analysis. Single cells were stained with the Fixable Viability Stain (FVS) 620, BV510 Rat Anti-Mouse CD45, APC-Cy7 Hamster Anti-Mouse CD3e, BB700 Rat Anti-Mouse CD4, fluorescein isothiocyanate (FITC) Rat Anti-Mouse CD8a, APC Hamster Anti-Mouse CD279 (PD-1), respectively. Cells were permeabilized using the eBioscience™ Set following the instruction protocol for intracellular staining. Subsequently, the cells were stained with BV421 Rat Anti-Mouse IFN-γ and PE/Cyanine7 anti-mouse Granzyme B. Finally, flow cytometry detection was conducted on LSRFortessa^™^ X-20 (Becton, Dickinson and Company, Franklin Lakes, NJ, USA).

### Statistical analysis

All data were shown as mean ± standard error of the mean (SEM). For normally distributed data, we used a two-tailed Student’s *t*-test to compare the statistical differences between any two groups. For three or more groups, one-way ANOVA was performed, followed by Bonferroni or Tamhane’s T2 post hoc tests. For data that were not normally distributed, the Wilcoxon test for two-group comparisons and the Kruskal–Wallis H test for three or more group comparisons were used. Statistical analyses were conducted with SPSS (Version 20) and GraphPad Prism (Version 9.0). *p* < 0.05 was considered to be statistically significant.

## Results

### DMF inhibits HCC progression in mice

**Fig. 1:**
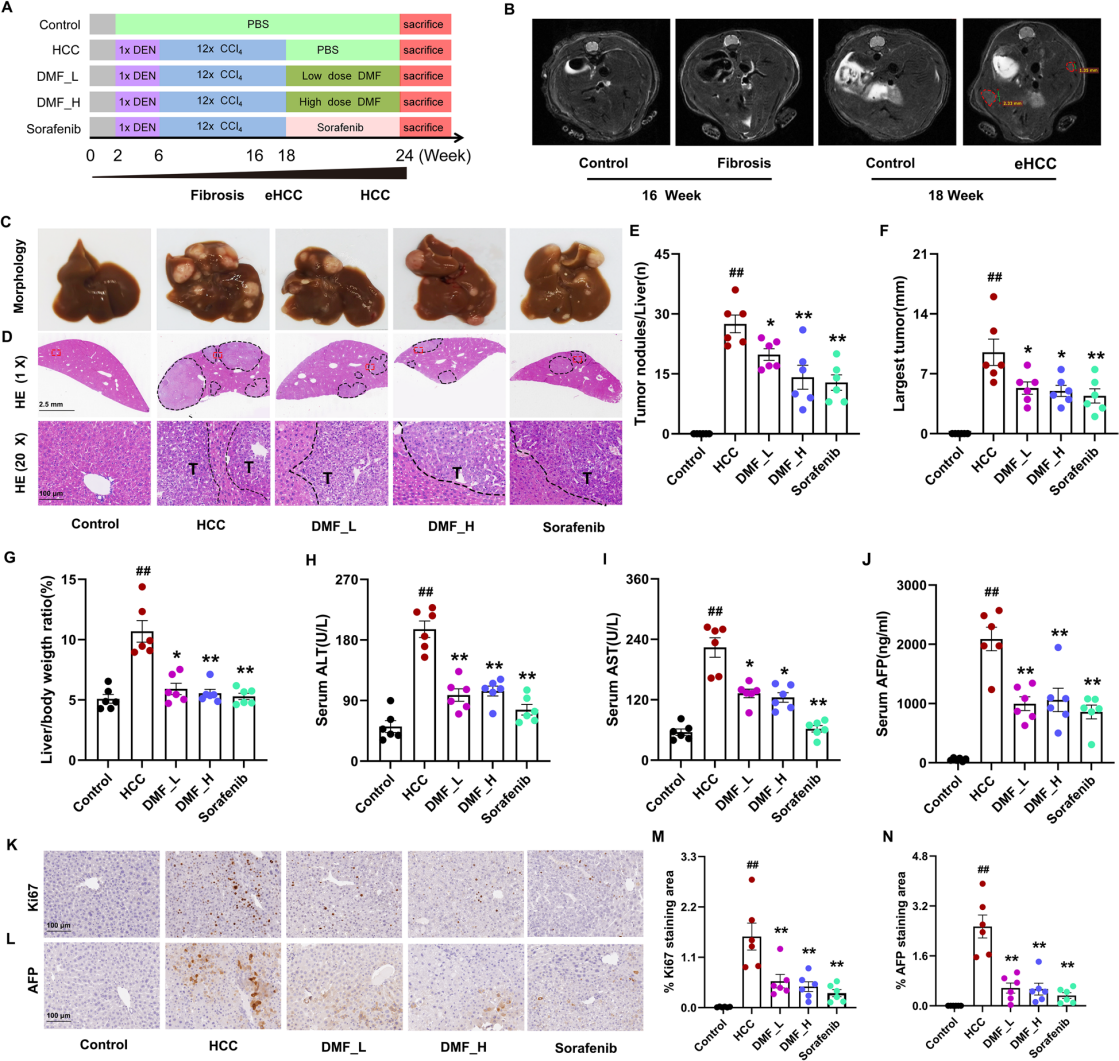
DMF inhibits DEN-CCl_4_-induced HCC progression. **A** The schematic of DEN/CCl_4_-induced HCC mouse model and the oral administration of DMF. For 24 consecutive weeks, mice were subjected to a single intraperitoneal (i.p.) injection of DEN (25 mg kg^−1^) at week 2, followed by 12 weekly i.p. injections with CCl_4_ starting at week 6. At week 18, mice received oral gavage treatment with either low-dose (40 mg kg^−1^; designated as DMF_L) or high-dose (80 mg kg^−1^; designated as DMF_H) DMF. Mice were sacrificed at week 24. **B** Representative MR images of liver. **C** Liver morphology. **D** HE staining. (10 × , top panel; 200 × , bottom panel). **E** Liver surface nodules ≥ 3 mm in diameter. **F** Maximum tumor diameter. **G** Liver/body weight ratio. **H** ALT levels. **I** AST levels. **J** Serum AFP levels. **K** IHC images of Ki67 in liver nodules (200 ×). **L** IHC images of AFP in liver nodules (200 ×). **M** Relative Ki67 staining area. **N** Relative AFP staining area. Data are shown as mean ± SEM, *n* = 6 per group. ^*^p < 0.05, ^**^p < 0.01 vs Control group, ^#^*p* < 0.05, ^##^*p* < 0.01 vs HCC group. eHCC: early-stage HCC

We explored the anti-tumor effects of DMF using a DEN/CCl_4_-induced HCC mouse model (Fig. 1A). Reportedly, mice develop fibrosis, followed by HCC, under the tumor-promoting effect of CCl_4_ [19]. Magnetic resonance imaging (MRI) showed liver fibrosis after 16 weeks, where substantial HCC development was observed after 18 weeks (Fig. 1B). After 24 weeks, all DEN/CCl_4_-treated mice developed typical HCC, as evidenced by liver morphology, HE staining, serum AFP levels, and IHC staining for AFP and Ki67 (Fig. 1C, D, J, K–N). Compared with the model group, DMF significantly reduced the tumor number, liver weight/body ratio, and maximum tumor diameter (Fig. 1E, F, G). The liver injury indicators serum ALT and AST decreased after DMF treatment (Fig. 1H, I). IHC analysis confirmed that the expression of the HCC malignancy indicators, AFP and Ki67 decreased significantly (Fig. 1K–N). DMF also reduced the serum AFP levels (Fig. 1J). Moreover, the efficacy of DMF in treating HCC was similar to that of sorafenib. These results show that DMF inhibits tumor progression in an HCC mouse model.

### DMF inhibits HCC partially dependent on *A. muciniphila*

Previous research has shown that gut microbiota dysbiosis is observed in liver fibrosis, cirrhosis, and HCC and that an imbalanced gut microbiota fuels the malignant progression of host HCC [28, 29]. We used 16S rRNA sequencing to detect changes in the abundance of the gut microbiota at different time points, thereby studying the role of DMF in the regulation of gut microbes. The results suggested that α-diversity, including Chao1, Simpson, and Shannon indices, was decreased at the fibrosis stage when the mice were 16 weeks old, with no statistical differences (*p* > 0.05) (Fig. S1A-F). However, the Chao1 indices (Fig. S1D) and Shannon (Fig. S1F) were significantly reduced at the HCC stage after 24 weeks. Notably, there was a decreased abundance of several probiotic bacteria (e.g., *Prevotellaceae_UCG_001*, *Lachnospiraceae_NK4A136_Group and A. muciniphila*) and an increased abundance of pathogenic bacteria, including *Parasutterella* (Fig. S1G).

**Fig. 2:**
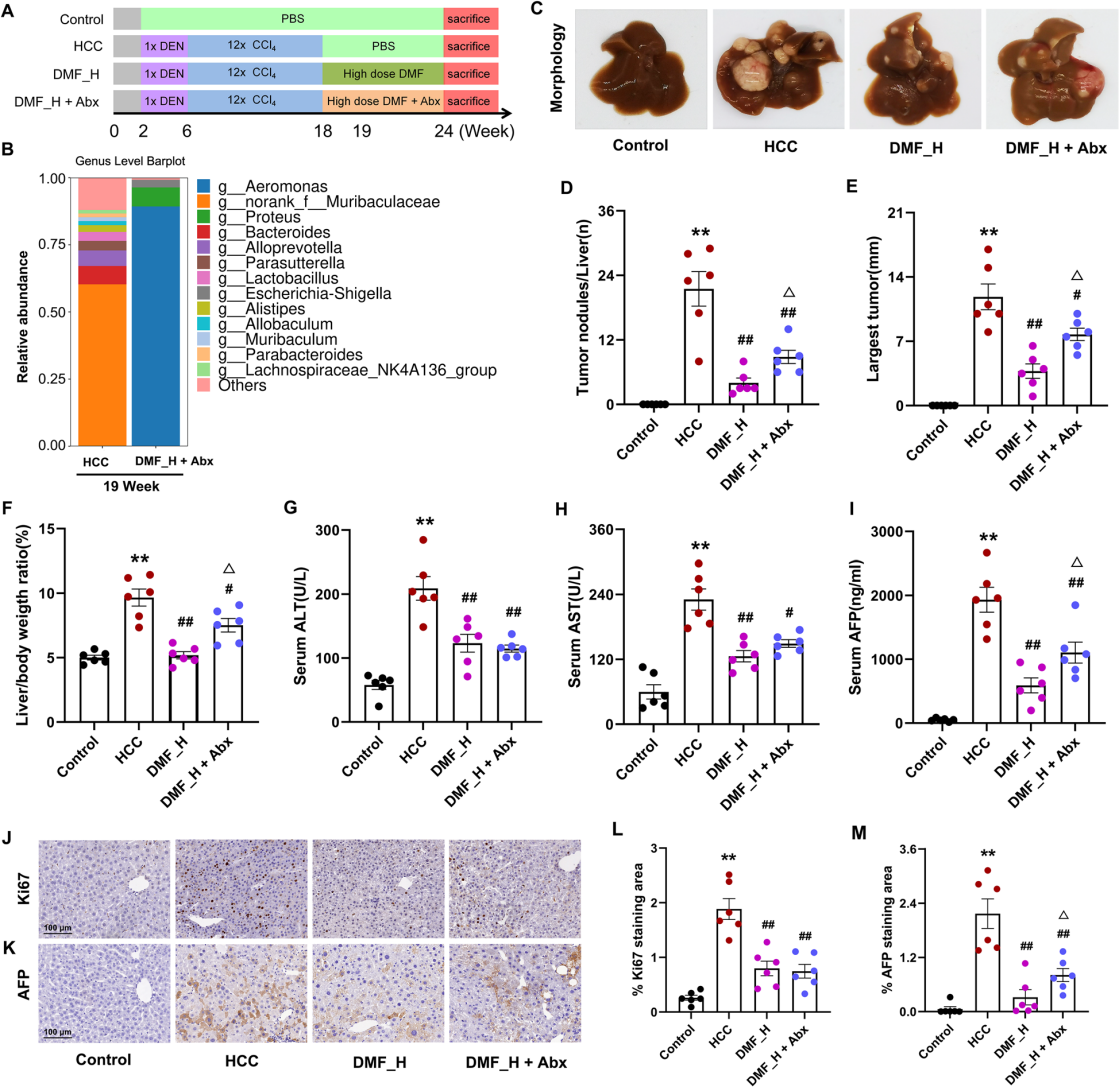
DMF inhibits HCC progression in an Abx-treated mice model. **A** Schematic representation of the HCC model and the administration of high-dose DMF orally, with or without Abx. At week 18, mice received a treatment of 80 mg kg^−1^ (DMF_H) via oral gavage. The mice was provided free access to water containing a cocktail of four non-absorbable and broad-spectrum antibiotics, starting at week 18 and continuing until sacrifice. **B** The genus levels after one week antibiotics cocktail. **C** Liver morphology. **D** Liver surface nodules ≥ 3 mm in diameter. **E** Maximum tumor diameter. **F** Liver/body weight ratio. **G** ALT levels. **H** AST levels. **I** Serum AFP levels. **J** IHC images of Ki67 in liver nodules (200 ×). **K** IHC images of AFP in liver nodules (200 ×). **L** Relative Ki67 staining area. **M** Relative AFP staining area. Data are shown as mean ± SEM, *n* = 6 per group. ^*^p < 0.05, ^**^p < 0.01 vs Control group, ^#^*p* < 0.05, ^##^*p* < 0.01 vs HCC group, ^△^*p* < 0.05, ^△△^*p* < 0.01 vs DMF_H group

We investigated whether DMF completely depended on the gut microbiota to explore its anti-tumor effect. Gut-sterilized HCC mice were established using oral antibiotics in drinking water [20]. High-dose DMF combined with an antibiotic cocktail was administered after 18 weeks (Fig. 2A). Feces were collected after 1 week of antibiotic cocktail treatment. We found that commensal bacteria were eliminated compared with the DMF_H group after 19 weeks (Fig. 2B). Antibiotics lowered the anti-tumor effect of DMF, confirmed by the significant increase in tumors, liver weight/body ratio, maximum tumor diameter, serum AFP levels (Fig. 2C–F, I), and AFP expression in tumor (Fig. 2K, M). These results show that DMF inhibition of HCC is partially dependent on the gut microbiota.

**Fig. 3:**
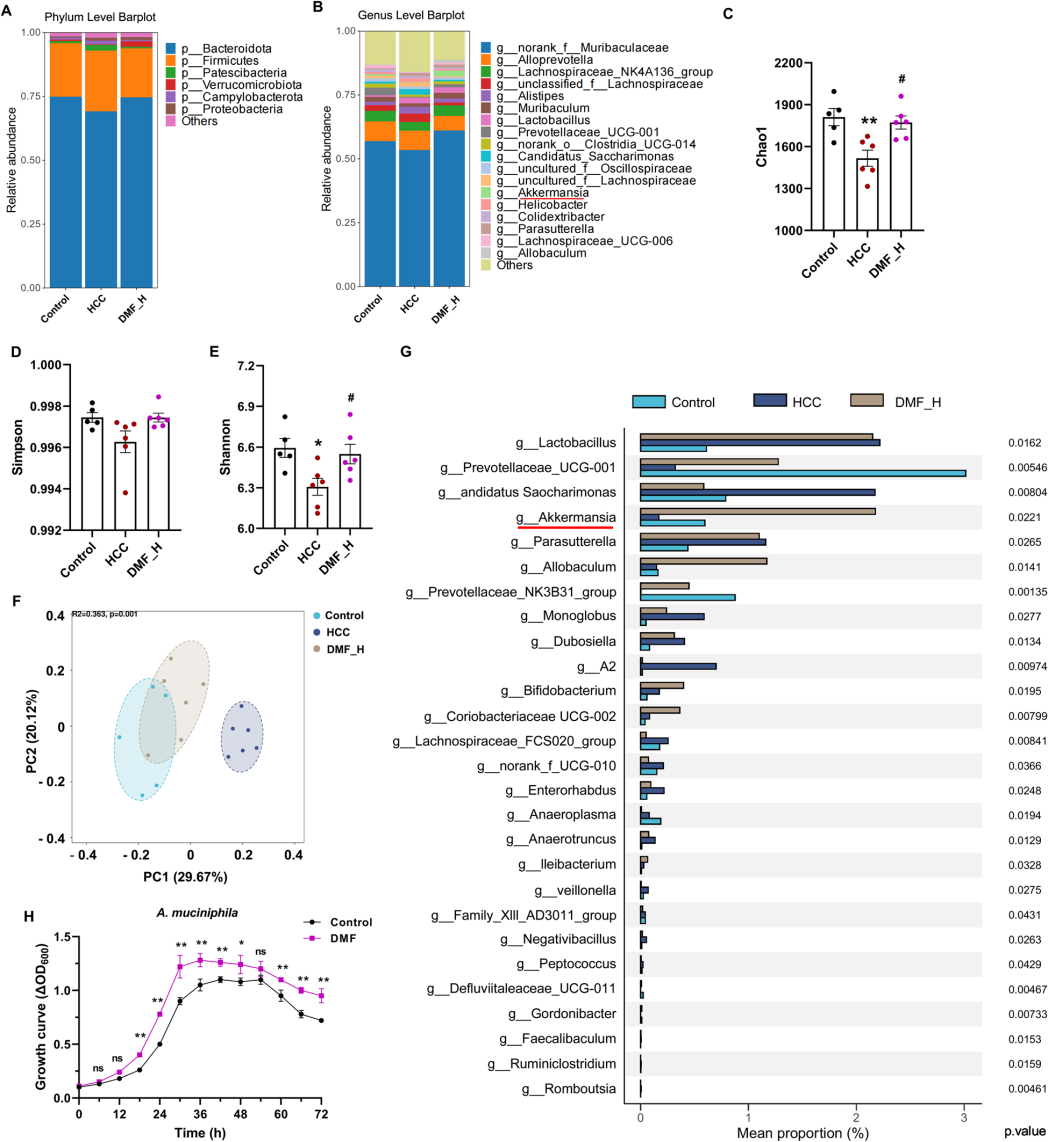
Alternation of the gut microbiota in HCC mice treated with DMF. **A** Bar chart illustrating microbial community composition categorized by phylum level. **B** The genus levels. **C**, **E** α-diversity assessments utilizing Chao1 (**C**), Simpson (**D**), and Shannon indices (**E**). **F** β-diversity evaluation conducted through PCoA based on Bray–curtis distance. **G** Identification of differentially abundant genera through multiple Kruskal–Wallis analyses comparing Control, HCC, and the DMF_H group. **H** Viable AKK number determined by measuring OD600. Data are shown as mean ± SEM, *n* = 5–6 per group in animal experiments, whereas *n* = 3 of independent in vitro experiments. ^*^p < 0.05, ^**^p < 0.01 vs Control group, ^#^*p* < 0.05, ^##^*p* < 0.01 vs HCC group

Subsequently, we explored which intestinal flora DMF regulated to exert its anti-tumor effect using 16S rRNA sequencing. Compared with the HCC group, DMF increased the richness and diversity of the gut microbiota in mice, as indicated by an increase in Chao1 and Shannon indices (Fig. 3A–E). The PcoA index was analyzed to evaluate the β-diversity. The control and HCC groups were significantly separated (*p* < 0.01), where the DMF group tended toward the control group (Fig. 3F), indicating that DMF could increase the species diversity and richness of mouse gut microbiota and restore the gut microbial composition to a near-normal level. Notably, there was a significant increase in the abundance of *A. muciniphila, Prevotellaceae-UCG-001,* and *NK3B31* and a decreased abundance of *Candidatus* and *Veillonella* (Fig. 3G). Additionally, DMF promoted *A. muciniphila* proliferation in vitro (Fig. 3H). These results show that DMF inhibits HCC, partially dependent on *A. muciniphila*.

### *A. muciniphila* treatment protects mice against HCC progression

**Fig. 4:**
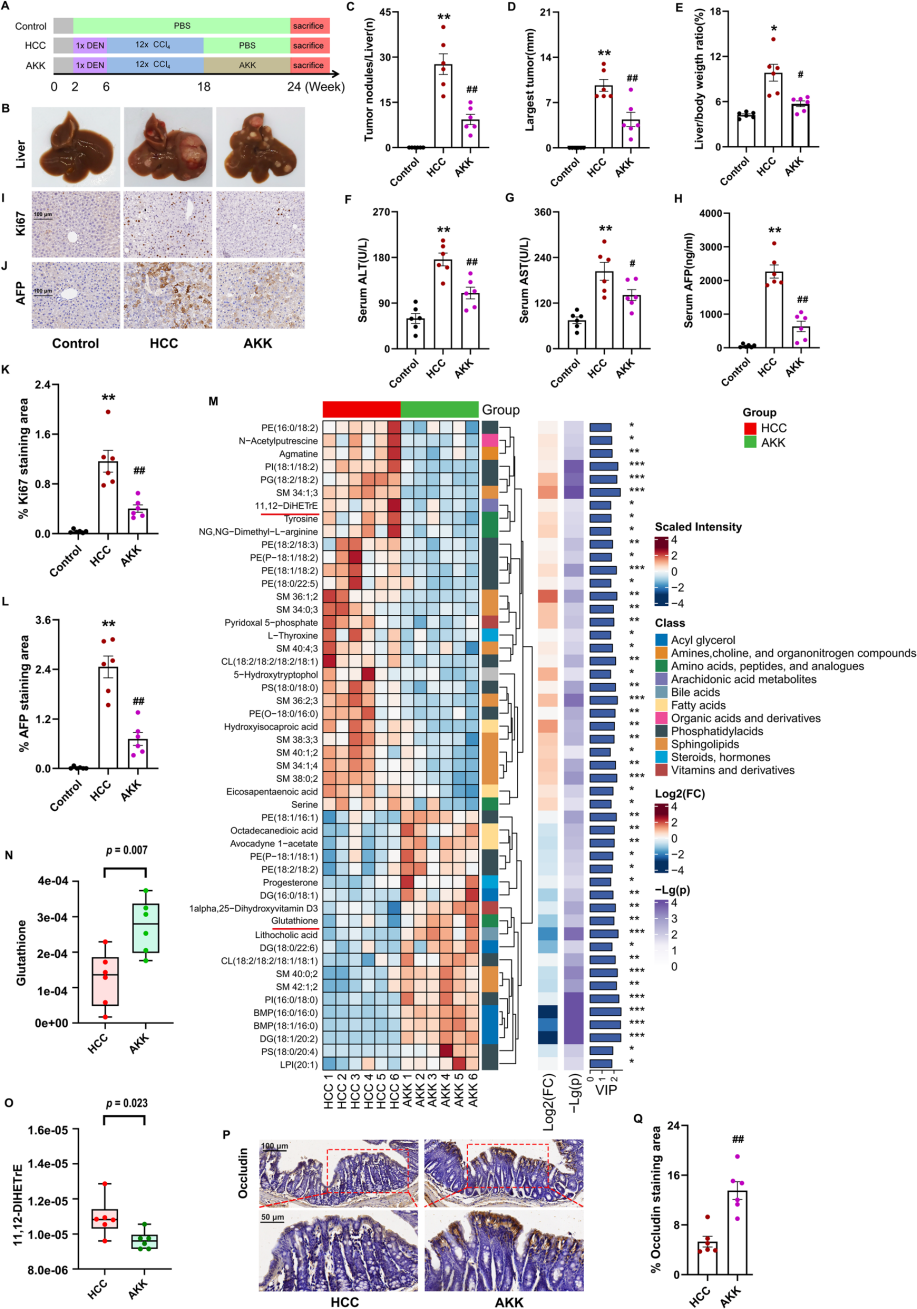
*A. muciniphila* supplement inhibits DEN-CCl_4_-induced HCC progression. **A** Schematic illustration of HCC model and oral administration of *A. muciniphila*. At week 18, mice were administered the *A. muciniphila* microbiota suspension orally twice a week for six weeks. **B** Liver morphology. **C** Liver surface nodules ≥ 3 mm in diameter. **D** Maximum tumor diameter. **E** Liver/body weight ratio. **F** ALT levels. **G** AST levels. **H** Serum AFP levels. **I** IHC images of Ki67 in liver nodules (200 ×). **J** IHC images of AFP in liver nodules (200 ×), **K** Relative Ki67 staining area. **L** Relative AFP staining area. **M** The metabolite heatmap of cecal contents by QMT1000. **N** The levels of glutathione. **O** The levels of 11,12-DIHETE. **P** IHC images of occludin in colon (top: 200 × , bottom: 400 ×). **Q** Relative occludin staining area. Data are shown as mean ± SEM, *n* = 6 per group. ^*^p < 0.05, ^**^p < 0.01 vs Control group, ^#^*p* < 0.05, ^##^*p* < 0.01 vs HCC group

Previous studies have shown that *A. muciniphila* regulates liver metabolism and antioxidant stress by reducing the metabolic endotoxin levels and improving intestinal barrier function in mice with metabolic dysfunction-associated fatty liver disease [30, 31]. Following the above results, HCC mice were administered active *A. muciniphila* to explore whether HCC progression could be inhibited (Fig. 4A). Interestingly, *A. muciniphila* significantly reduced the tumor number, liver weight/body ratio, maximum tumor diameter, and serum ALT, AST, and AFP levels (Fig. 4B–H). Additionally, AFP and Ki67 expression in the tumors significantly decreased (Fig. 4I–L). QMT1000 was used to explore the effects of *A. muciniphila* on the metabolite profile of cecal contents in HCC mice. Supplementation with *A. muciniphila* enhanced the antioxidant stress by upregulating glutathione and downregulating 11,12-DIHETE (Fig. 4M, O). Moreover, *A. muciniphila* directly upregulated occludin expression (Fig. 4P, Q), thus restoring the impaired gut barrier function.

### ***DMF inhibits the HCC progression by enhancing CD8***^+^***T cells-mediated anti-tumor immunity***

An untargeted serum metabolomic analysis was conducted. The overall differences between the groups (HCC and DMF_H) were significant, as shown by the distances between the samples from the two groups (Fig. S3A, B). The metabolites with the highest upregulation was DMF (Fig. S3C, D). These results show that DMF inhibition of HCC is partially dependent on the gut microbiota, and there exist additional mechanisms underlying its anti-tumor effect following its absorption into the bloodstream.

**Fig. 5:**
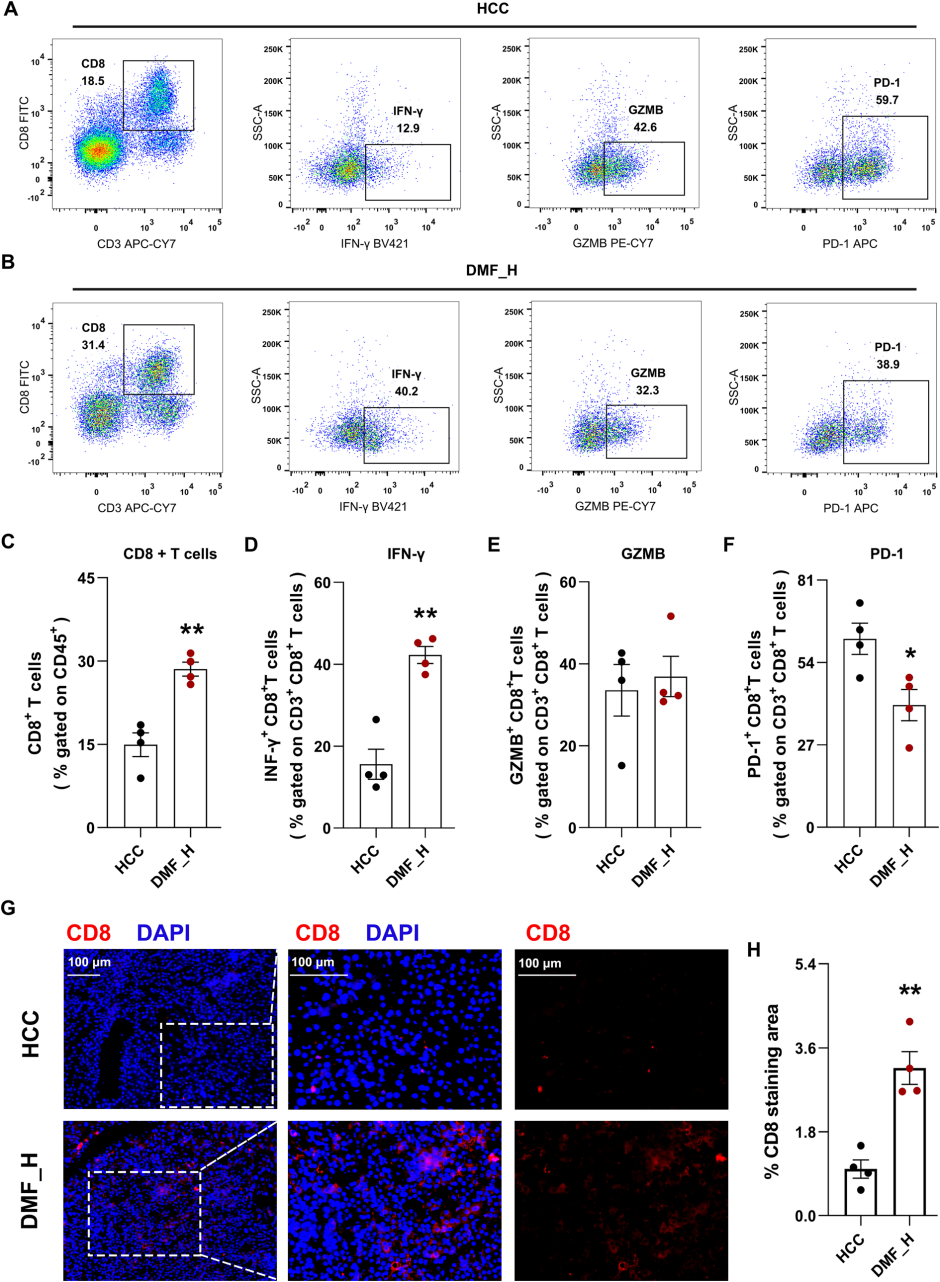
The effect of DMF on anti-tumor function of CD8^+^ T cells in HCC mice. **A**, **B** The percentage of CD8^+^ T cells was detected by flow cytometry in the tumor tissues **A**, and the levels of IFN-γ, GZMB and PD-1 in CD8^+^ T cells in HCC and DMF_H group (**B**). **C** Liver CD8^+^ T cells as percentage of liver CD45^+^ cells. **D** Liver IFN-γ^+^ CD8^+^ T cells as percentage of total liver CD3^+^ CD8^+^ T cells. **E** Liver GZMB^+^ CD8^+^ cells as percentage of total liver CD3^+^ CD8^+^ T cells. **F** Liver PD-1^+^ CD8^+^ cells as percentage of total liver CD3^+^ CD8^+^ T cells. **G** Immunofluorescence staining images of liver sections stained with anti-CD8 (red) and DAPI (blue), left, middle and right panels: are 100 × , 200 × , 200 × , respectively. **H** Relative CD8 staining area. Data are shown as mean ± SEM, *n* = 4 per group. ^*^p < 0.05, ^**^p < 0.01 vs HCC group

In the current study, we initially focused on CD8⁺ T cells in the tumor microenvironment because their dysfunction is a well-established hallmark of immunosuppression in tumor type. Thus, we detected the infiltration of CD8^+^ T cells and the expression of several effectors. The results showed that DMF treatment significantly increased CD8^+^ T cell infiltration (Fig. 5A–C). Importantly, DMF enhanced the anti-tumor effect of CD8^+^ T cells as interferon-γ (IFN-γ) increased and programmed cell death protein 1 (PD-1) decreased (*p* < 0.05) (Fig. 5A, B, C, F). However, there was no statistical difference in granzyme B (GZMB) levels between the DMF_H and HCC groups (*p* > 0.05) (Fig. 5A, B, E). Next, we confirmed CD8^+^ T cell infiltration. Immunofluorescence staining showed that DMF increased infiltrating CD8^+^ T cells into the tumor microenvironment (TME), following the flow cytometry results (Fig. 5G, H). These results revealed that DMF can effectively inhibit HCC progression by increasing infiltration and enhancing the killing function of CD8^+^ T cells.

### ***DMF enhances the infiltration effect of CD8***^+^***T cells by targeting CCL2 in HCC cells***

**Fig. 6:**
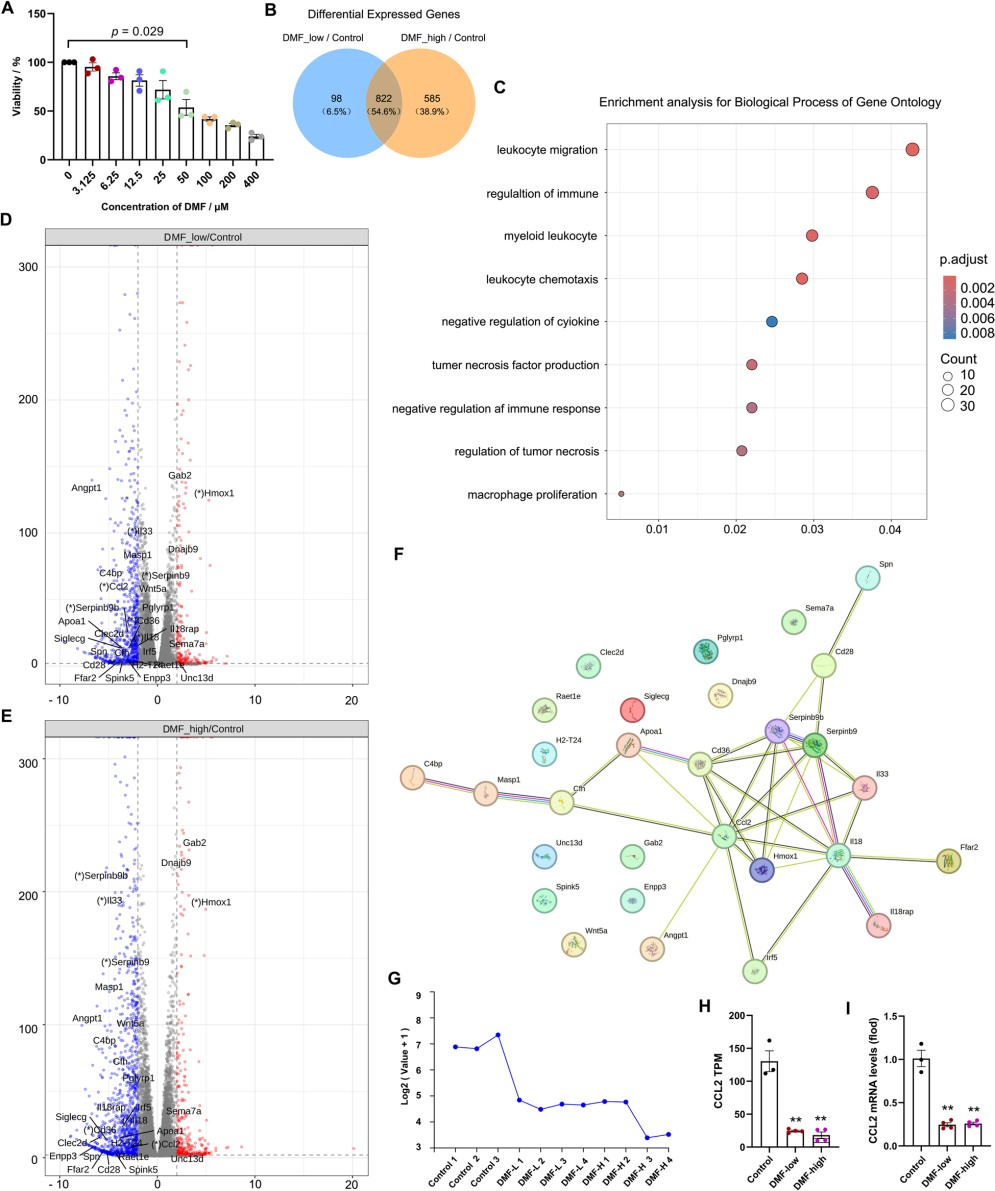
Transcriptome analysis results. **A** Cell viability assessed across different concentrations of DMF treatment using CCK8 assay. **B** Venn diagram illustrating the identification of 882 differentially expressed genes between the groups treated with low-dose and high-dose DMF compared to control. **C**, **D** Venn diagrams displaying volcanic plots for the identified genes from both low-dose (**C**), and high-dose (**D**) treatments against control; upregulated genes are represented in light red while downregulated genes appear in dark blue. **E** Gene Ontology analysis. **F** PPI network plot focusing on CCL2. **G** Line chart representing average CCL2 expression levels in Hepa1-6 cells. **H**, **I** Assessment of CCL2 expression through PTM (**H**), and RT-PCR (**I**). Data shown are the mean ± SEM, *n* = 3–4, three or four independent in vitro experiments. ^**^p < 0.01 vs Control group

To understand how DMF modulates the TME, we prioritized analyzing the crosstalk between tumor cells and CD8⁺ T cell exhaustion because our primary focus was to first clarify DMF’s direct effect on tumor cells after it is absorbed into the bloodstream. Therefore, we treated Hepa1-6 cells with DMF to establish a mouse hepatoma cell line in vitro. CCK8 assay showed the viability of 50 μM DMF was 53.7% and significantly inhibited the cell proliferation (*p* = 0.029) (Fig. 6A). Thus, low dose DMF (25 µM, DMF_L), high dose DMF (50 µM, DMF_H) were used, and bulk RNA-seq analysis was conducted. For the differential gene analysis, with a Qvalue < 0.01 and log2 fold change > 2 or < -2, 920 differentially expressed genes were screened between DMF_low and control (215 upregulated/705 downregulated), and 1407 genes were screened between DMF_high and control (326 upregulated/1081 downregulated) (Fig. 6B). Moreover, there were 822 overlapping differentially expressed genes between the two groups.

Clinical studies of HCC have been dominated at all stages by combination therapies based on immune ICIs [32]. Therefore, we focused on the signaling pathways involved in the changes in immune regulatory functions induced after treating the hepa1-6 cell lines with DMF. Firstly, Gene ontology (GO) analysis identified 214 pathways. Through overrepresentation analysis, nine immune-related GO pathways were highlighted using enrichment plots (Fig. 6C, Supplementary Table 3). Furthermore, 29 genes were involved in regulating these immune effector processes (Fig. 6D, E), and their protein–protein interaction relationships were identified using STRING database (Supplementary Table 4). The top seven genes in terms of node degree were selected as the core genes, with CCL2 having the greatest network contribution (degree = 10) (Fig. 6F). Notably, the reverse transcriptase-PCR of CCL2 was in accordance with the transcriptome sequencing results (*p* < 0.01, Fig. 6G–I). The results indicate that CCL2 is an important target gene of DMF.

### DMF decreases CCL2 expression by suppressing the NF-κB pathway

**Fig. 7:**
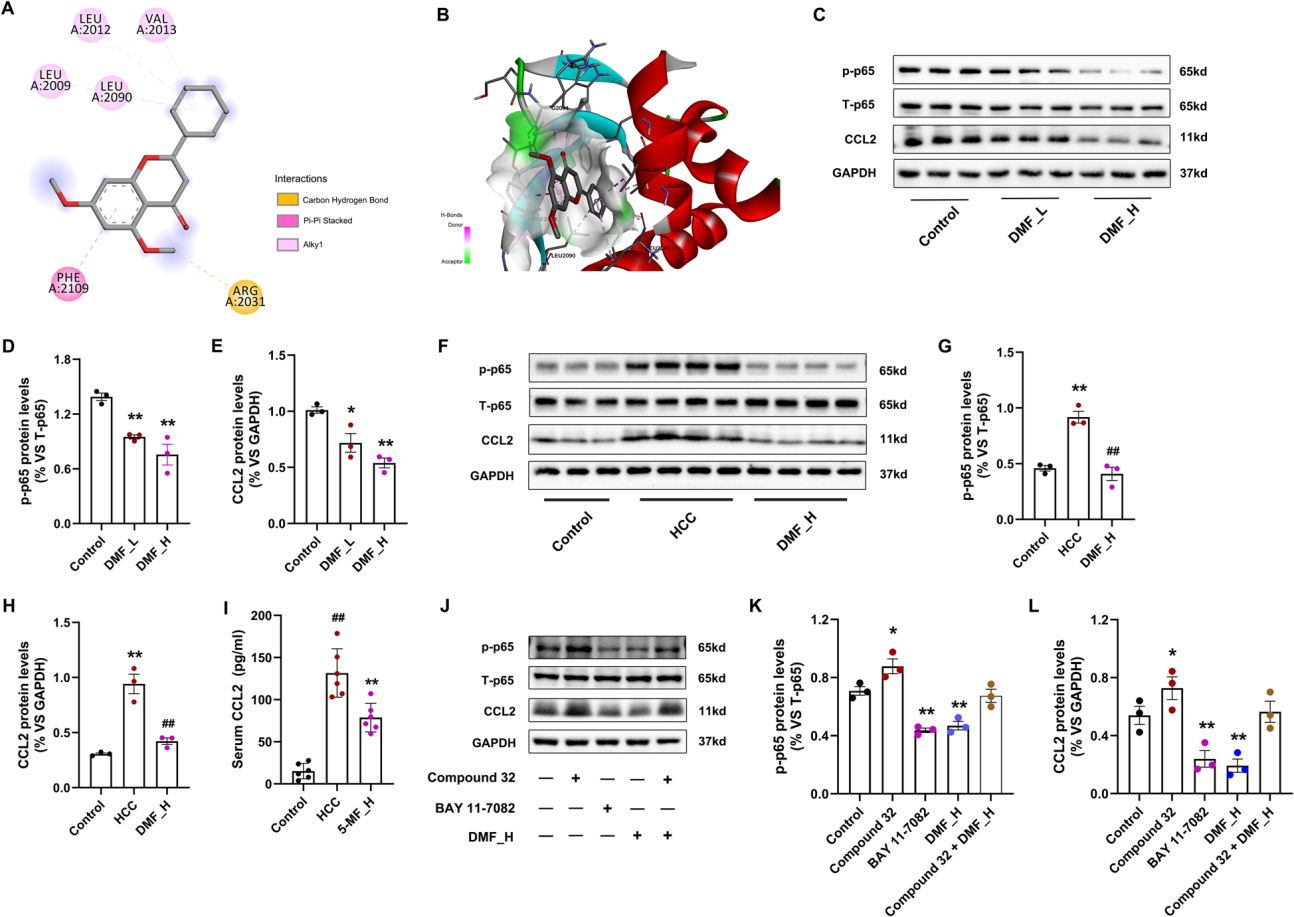
DMF dose-dependently inhibits the constitutive activation of the NF-κB pathway. **A**, **B** Representative images illustrating the interaction poses of DMF and NF-κB p65 in both 2D (**A**), and 3D formats (**B**). **C** Immunoblots of p-p65, T-p65, CCL2, and GAPDH in Hepa1-6 cells. Control group cells were incubated with DMEM containing 10% FBS for 24 h (Control group), or treated with low dose DMF (25 µM, referred to DMF_L) or high dose DMF (50 µM, DMF_H) for 24 h. **D**, **E** The relative protein expression of p-p65 compared to T-p65 (**D**), and CCL2 (**E**) compared with GAPDH, (*n* = 3, three independent experiments in vitro experiments). **F** Immunoblots of p-p65, T-p65, CCL2 and GAPDH in the mouse HCC tissue. **G** The relative protein expression of p-p65. **H** The relative protein expression of CCL2, (*n* = 3–4 per group). **I** Serum CCL2 levels, (*n* = 6 per group). **J** Immunoblots of p-p65, T-p65, CCL2 and GAPDH in Hepa1-6 cells treated with either the NF-κB agonist Compound 32 or inhibitor Bay 11–7082, or combined with treatment using either Compoud 32 or high-dose DMF, respectively. **K**, **L** The relative protein expression of p-p65 (**K**), and CCL2 (**L**), (*n* = 3). Data shown are the mean ± SEM. ^*^p < 0.05, ^**^p < 0.01 vs Control group, ^#^*p* < 0.05, ^##^*p* < 0.01 vs HCC group

Inflammatory signaling pathways are involved in developing cancer. Activation of NF-κB p65, STAT3, and ERK is important for upregulating CCL2 expression and promoting inflammation and cancer [33]. The combination affinity of DMF with NF-κB p65, STAT3, and ERK was 8.4, 9.9, and 7.6, respectively, and the representative image of the docking results of DMF with NF-κB p65 was shown (Fig. 7A, B), STAT3, and ERK (Fig. S3A-D). Next, we confirmed whether NF-κB p65, STAT3, and ERK pathways are regulated by DMF in Hepa1-6 cells. Western blot showed that only phospho-NF-κB p65 (p-p65) was significantly dose-dependently inhibited (Fig. 7C–E), but p-STAT3 and p-ERK1/2 levels were unaffected (Fig. S3A-G). Moreover, a significant decrease in p-p65 and serum CCL2 was observed in mouse HCC treated with DMF (Fig. 7G–H). Finally, we treated Hepa1-6 cells with NF-κB agonist Compound 32 and inhibitor Bay 11-7082. We discovered that both DMF and Bay 11-7082 inhibited the activation of NF-κB signaling, and Compound 32 treatments significantly reduced the inhibitory effect of DMF (Fig. 7I–K). These results show that DMF decreases CCL2 expression by suppressing the constitutive activation of the NF-κB pathway in HCC cells.

## Discussion

HCC is a complex multifactorial process, and over 80% of patients develop either fibrosis or cirrhosis [2]. Specifically, the liver is an immune organ that forms a unique immunosuppressive microenvironment to avoid liver damage caused by various pathogens and intestinal microorganisms through the gutliver axis [34]. In this study, a DEN/CCl_4_-induced HCC mouse model was developed with fibrotic livers based on the characteristics of the natural occurrence of human HCC. Our results showed that gut microbiota dysbiosis was linked to a decreased abundance of several probiotic bacteria and an increased abundance of pathogenic bacteria (Fig. S1), which is similar to that observed in human liver fibrosis, cirrhosis, and HCC [35].

Flavonoids, a class of compounds with a 2-phenyl chromone structure, mainly include six categories: flavonols, flavanols, flavanones, flavones, isoflavones, and anthocyanins, which can alter the composition of the gut microbiota [36]. We discovered that DMF, a flavonoid, could restore the gut microbial composition with an upregulated abundance of probiotic bacteria, including *A. muciniphila*, and a decreased abundance of pathogenic bacteria. The deficiency or reduction of *A. muciniphila* is related to a variety of diseases, such as obesity, diabetes, fatty liver disease, inflammation, as well as responses to cancer immunotherapy, through specialization of its mucin metabolism, short-chain fatty acid production, and regulation of the decline of other probiotic bacteria [37]. Supplementation with *A. muciniphila* restored the redox balance as it increased glutathione and decreased 11,12-DIHETE (Fig. 4N, O). These results are similar to findings in a previous study that showed an increase in glutathione content in the livers of mice treated with *A. muciniphila* [31]. Glutathione, an endogenous antioxidant, can alleviate oxidative stress and inflammatory response caused by viral infections and protect liver cells from damage, thereby inhibiting the progression of liver inflammation and fibrosis [38]. 11,12-DIHETE is generated from arachidonic acid through oxidation, followed by hydrolysis, and participates in the stress and injury response, and is significantly increased in nonalcoholic steatohepatitis or acutely decompensated cirrhosis [39, 40]. *A. muciniphila* restores the intestinal barrier and slows intestinal inflammation by increasing the protein levels of occludin in the colon, reducing the entry of lipopolysaccharide (LPS) into the bloodstream. The results of further studies showed that DMF promoted *A. muciniphila* proliferation in vitro (Fig. 3H). However, some studies have found that pasteurized *A. muciniphila* still improves host health, including enhancing immune response by a lipid from *A. muciniphila*'s cell membrane [41], or improving metabolic disorders by an outer membrane protein of *A. muciniphila* [42]. We focused on regulating DMF using a living *A. muciniphila,* which protected mice against HCC progression, avoiding the use of supplementation with pasteurized *A. muciniphila*; however, evaluating the potential anti-tumor effect is impossible. These results showed that DMF alters the diversity of the *A. muciniphila* in mice and inhibits HCC progression.

The results of the combined antibiotic treatment showed that the anti-HCC effect of DMF did not entirely rely on specific gut microbiota (Fig. 2). This finding shows that DMF could be absorbed into the bloodstream before eliciting its anti-tumor effects. We further confirmed this result using untargeted serum metabolomic analysis (Fig. S2). In the current study, we initially focused on CD8⁺ T cells because their dysfunction is a well-established hallmark of immunosuppression in tumor type. HCC can be classified into two types based on the status of immune cell infiltration in the TME: immune (a ‘hot tumor’) and immune-exempt (a ‘cold tumor’) [43]. The main characteristic of a ‘cold tumor’ is the lack of CD8^+^ T cell infiltration within the tumor. Nevertheless, a ‘hot tumor’ is characterized by a higher degree of immune cell infiltration. The immune type can be further subdivided into the immune-active subtype (which is rich in IFN signaling transduction and T cell cytotoxicity markers) and the immune-exhausted subtype (which shows a lower expression of IFN-γ and cytotoxicity markers, with a high expression of programmed death-ligand 1 (PD-L1) and its receptor, PD-1). Forming an immunosuppressive microenvironment in the liver is closely related to a low response rate of HCC immunotherapy [32]. DMF can considerably increase the infiltration and improve the killing function of CD8^+^ T cells, indicated by the increase of IFN-γ and the decrease of PD-1 (Fig. 5D, F). Therefore, DMF treatment would benefit the majority of the patients by reshaping the immunosuppressive microenvironment of HCC.

We prioritized analyzing the crosstalk between tumor cells and CD8⁺ T cell exhaustion because our primary focus was to first clarify DMF’s direct effect on tumor cells following its absorption into the bloodstream. Importantly, transcriptome analysis, docking assay, and western blotting showed that DMF decreased CCL2 expression in HCC cells. The CCL2-CD8^+^ T cell axis has been widely reported and demonstrated. When CCL2 was knocked down in tumor cells or a CCL2-neutralizing antibody was used, the infiltration of CD8^+^ T cells was promoted [44–46]. Thus, as long as CCL2 is intervened, CD8^+^ T cells can be regulated. We focus on the upstream signaling pathways that mediate CCL2 expression, and found that DMF decreases CCL2 expression levels by suppressing constitutive NF-κB pathway activation, but not the ERK1/2 or STAT3 pathways, which were activated in the tumors of HCC mice (Fig. S3H–J). NF-κB consists of five subunits: p50, p52, RelA (p65), c-Rel, and Rel B, and exists in an inactive form in the cytoplasm. However, multiple factors in the HCC microenvironment can trigger NF-κB p65 activation (Fig. 7F, G). Activated NF-κB p65 translocates into the nucleus, where it binds to κB sites and regulates the transcription of target genes; this has a profound impact on tumor growth and progression [47]. Due to the constraints of research cycle, and experimental resources, we were unable to directly adopt the CCL2 neutralizing antibodies or CCL2 knockout mice to verify its function in this study. Additional investigations may be needed in future work to complete the functional validation aspect, which is essential for strengthening the translational relevance of this research.

## Conclusion

In this study, our findings revealed that DMF inhibited HCC progression in mice. The anti-tumor effect was mediated by increasing the species diversity and the richness of the mice gut microbiota, especially for *A. muciniphila,* and decreasing CCL2-dependent immunosuppression through the NF-κB pathway. These results showed that DMF and *A. muciniphila* may be novel therapeutic agents for HCC.

## Funding

This work was supported by the National Natural Science Foundation of China (grant numbers 82405072, 82274286 and 82304927); Joint Funds of Basic and Applied Basic Research Fund of Guangdong (grant number 2023A1515110041); China Postdoctoral Science Foundation (grant number 2023M741596).

## Supplementary Information

Supplementary Material 1.

## Data Availability

All the data for this study are available from the corresponding author upon rational request.

## Abbreviations

Abx: Antibiotic cocktail
AFP: Alpha-fetoprotein
AKK: *Akkermansia muciniphila*
ALT: Alanine aminotransferase
AST: Aspartate aminotransferase
CCL2: Chemokine (C–C motif) ligand 2
CCl_4_: Carbon tetrachloride
DEN: Diethylnitrosamine
DMEM: Dulbecco’s modified eagle’s medium
DMF: 5,7-Dimethoxyflavone
ERK: Extracellular regulated protein kinases
FBS: Fetal bovine serum
HCC: Hepatocellular carcinoma
HE: Hemataxylin and eosin
ICIs: Immune checkpoint inhibitors
IF: Immunofluorescence
IFN-γ: Interferon-γ
IHC: Immunohistochemical
MRI: Magnetic resonance imaging
NF-κB: Nuclear factor kappa-B
PBS: Phosphate buffered saline
PD-1: Programmed cell death protein 1
PD-L1: Programmed death-ligand 1
STAT3: Signal transducer and activator of transcription 3
TME: Tumor microenvironment
