# Supplementary material for: 5,7-dimethoxyflavone inhibits hepatocellular carcinoma progression via increasing intestinal Akkermansia muciniphila and hepatic CD8+ T cell infiltration
Source: Chin Med. 2025 Oct 8;20:170. doi: 10.1186/s13020-025-01233-8 (PMC12505807; doi:10.1186/s13020-025-01233-8)
Supplement: Supplementary file 1 — Supplementary Material 1. [file 13020_2025_1233_MOESM1_ESM.docx]

**5,7-Dimethoxyflavone inhibits hepatocellular carcinoma progression via increasing intestinal *Akkermansia muciniphila* and hepatic CD8^+^ T cell infiltration**

Weicong Chen^1,^ ^2✝^, Changshun Liu^2✝^, Xiao Li^3✝^, Xuemei Yang^1^, Yang Liu^2^, Mengchen Qin^2^, Wentao Jiang^2^, Yiqin Wang^2^, Haitao Sun^2^, Guohuan Li^1*^, Bin Wen^4*^, Songqi He^2*^

**Supplementary Figures S1-S3**

**
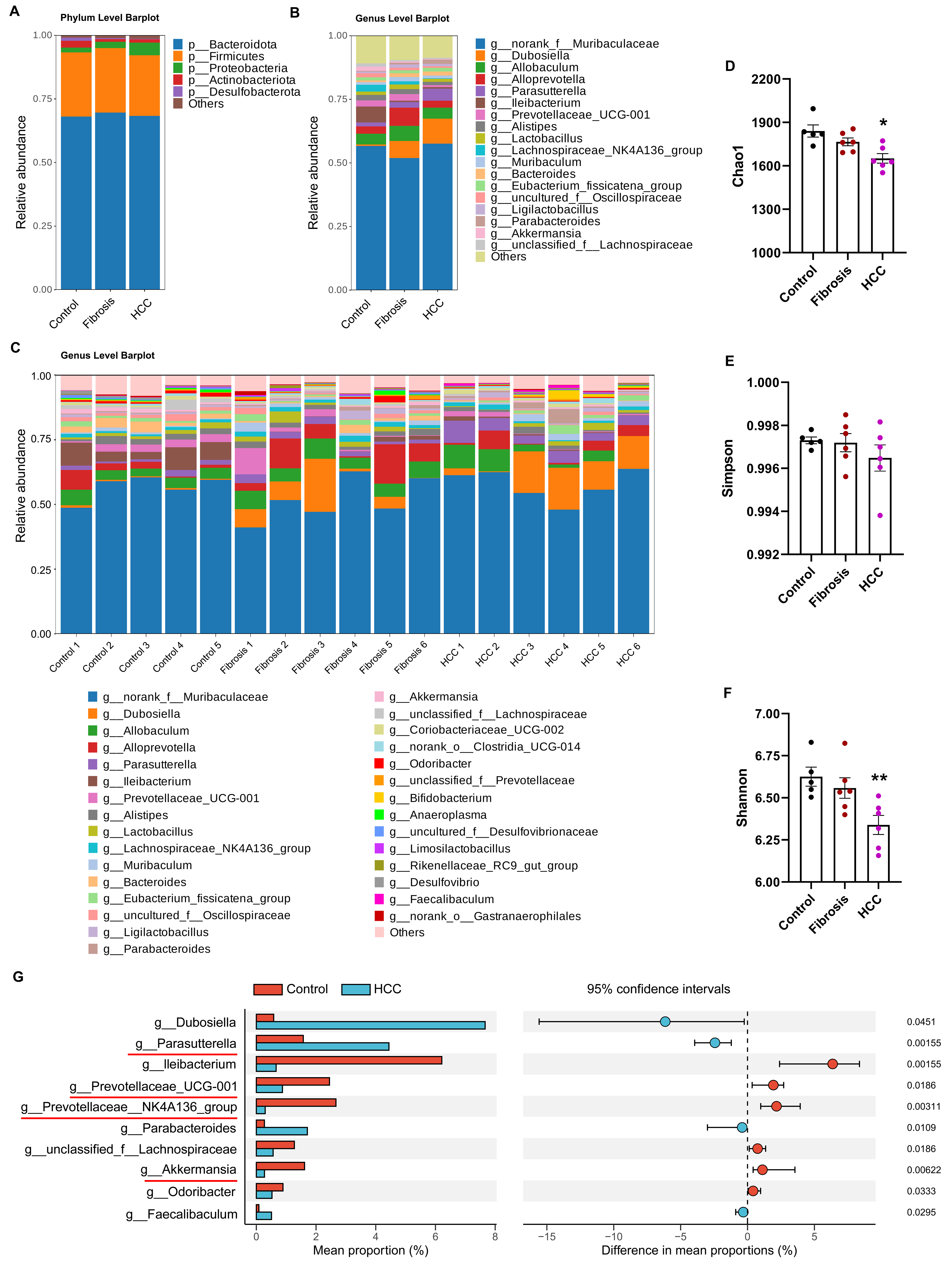
**

**Figure S1.** Alternation of the gut microbiota in HCC mice at different stage. **(A)** Bar chart of the microbial community at the phylum level. **(B-C) The genus levels. (D-F) Analysis of** α-diversity analysis by Chao1 **(D)**, Simpson **(E)**, and Shannon index **(F)**. **(G)** The top ten of differentially abundant genera using Wilcoxon test between the Control and HCC group Data are shown as mean ± SEM, *n* = 5-6 per group. ^*^*p*＜0.05, ^**^*p* < 0.01 vs Control group.

**
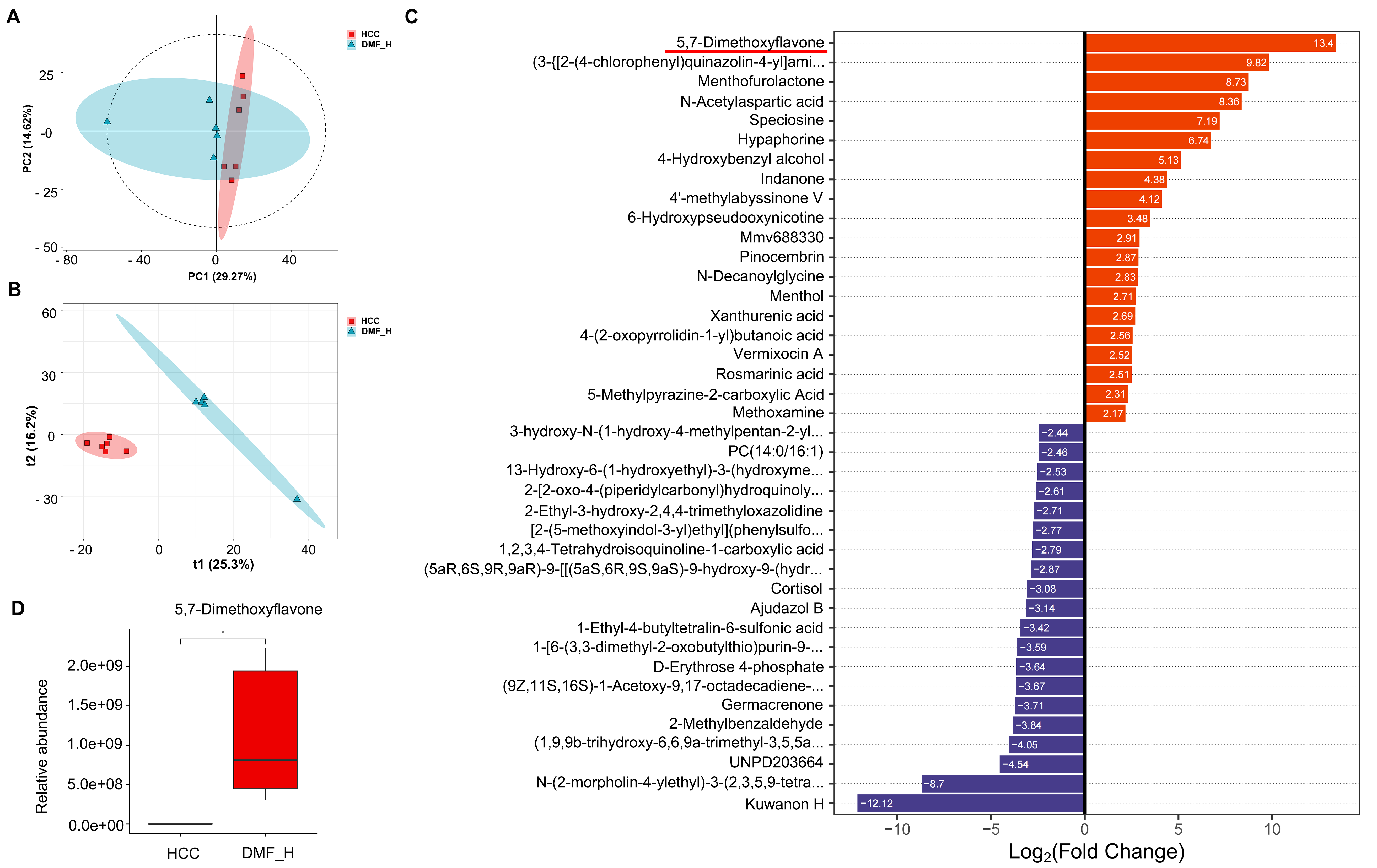
**

**Figure S2.** The untargeted serum metabolomics analysis. **(A)** Principal component analysis (PCA) of the characteristics of serum metabolomics. **(B)** Partial least squares discriminant analysis (PLS-DA). **(C)** The fold change bar of serum metabolite. **(D)** The relative levels of 5,7-Dimethoxyflavone. Data are shown as mean ± SEM, *n* = 5-6 per group. ^*^*p* < 0.05 vs HCC group.


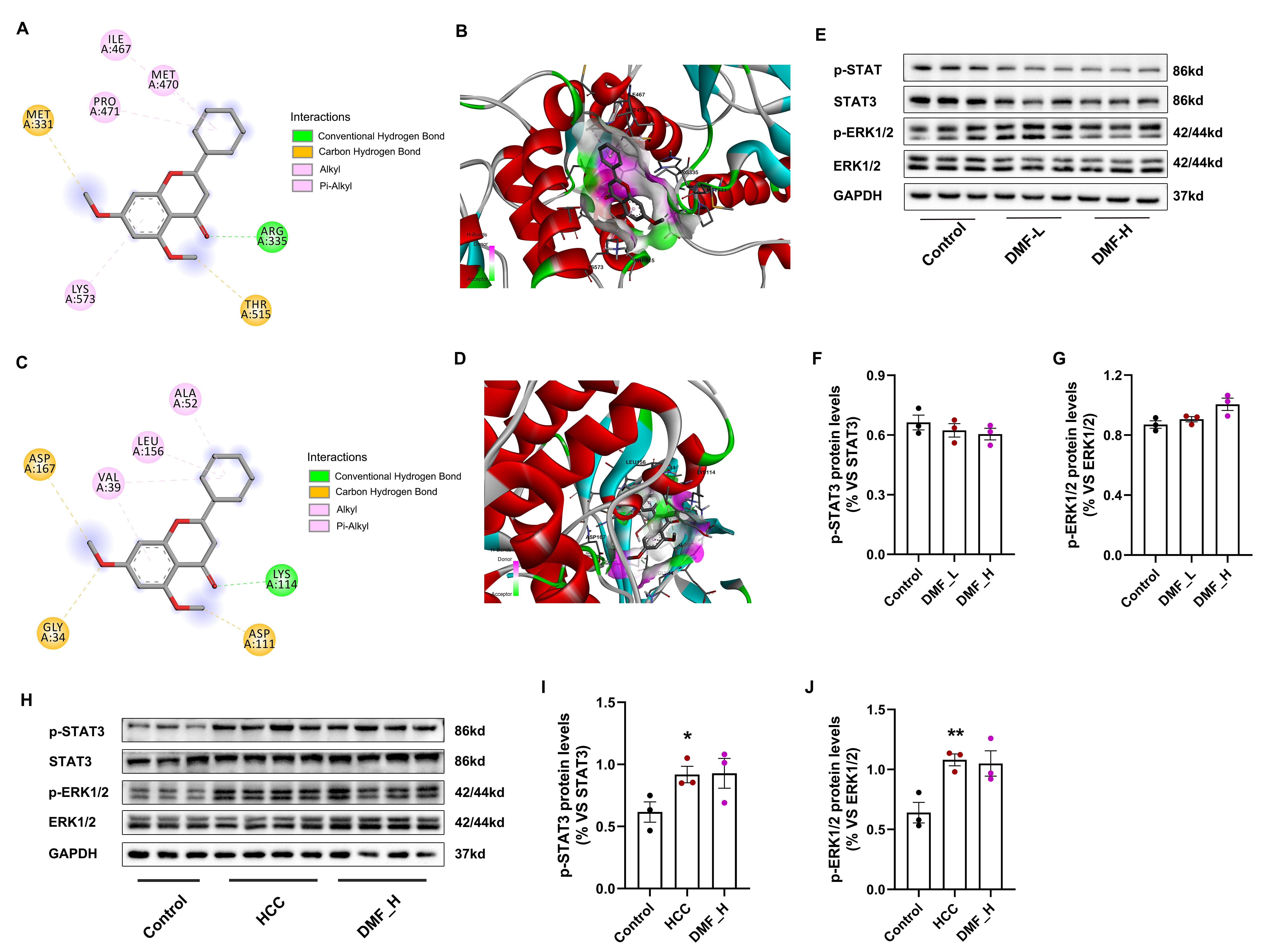


**Figure S3.** Effect of DMF on STAT3 and ERK pathway. **(A-B)** The 2D and 3D representative images of the interaction poses of DMF and STAT3. **(C-D)** The 2D and 3D representative images of the interaction poses of DMF and ERK. **(E)** Immunoblots of p-STAT3, STAT3, p-ERK1/2, ERK, and GAPDH in Hepa1-6 cells. The control group cells were incubated with DMEM containing 10 % FBS for 24 h (Control group), or treated with low dose DMF (25 µM, defined as DMF_L) or high dose DMF (50 µM, DMF_H) for 24 h. **(F-G)** The relative protein expression of p-STAT3 compared with STAT3, and p-ERK1/2 compared with ERK, (*n* = 3, three independent experiments in vitro experiments). **(H)** Immunoblots of p-STAT3, STAT3, p-ERK1/2, ERK, and GAPDH in the mouse HCC tissue. **(I-J)** and the relative protein expression of p-STAT3 and p-ERK1/2, Data shown are the mean ± SEM, *n* = 3-4 per group. ^*^*p* < 0.05, ^**^*p* < 0.01 vs Control group.

**Supplementary Tables S1-S4**

**Table S1**  **Antibodies used in this study.**

| Antibodies to: | Cat No. | Source | Host |
| --- | --- | --- | --- |
| AFP | AF5134 | Affinity | Rabbit |
| Ki67 | AF0198 | Affinity | Rabbit |
| Occludin | A5381 | Bimake | Rabbit |
| MCP-1 (CCL2) | Ab308522 | Abcam | Rabbit |
| GAPDH | 60004-1 | Proteintech | Mouse |
| STAT3 | F0200 | Bimake | Mouse |
| Phospho-Stat3 (Tyr705) | 4093 | CST | Rabbit |
| p44/p42 MAPK (Erk1/2) | F0002 | Bimake | Rabbit |
| Phospho-Erk1(T202/Y204)/Erk2 (T185/Y187) | A5036 | Bimake | Rabbit |
| NF-kB p65 | AF5006 | Affinity | Rabbit |
| Phospho-NF-kB p65 (Ser536) | AF20006 | Affinity | Rabbit |
| CD8a | 14-0081-82 | Invitrogen | Rat |
| BV510 Rat Anti-Mouse CD45(30-F11) | 563891 | BD Pharmingen | Rat |
| APC-Cy7 Hamster Anti-Mouse CD3e(145-2C11) | 557596 | BD Pharmingen | Hamster |
| BB700 Rat Anti-Mouse CD4 | 566407 | BD Pharmingen | Rat |
| FITC Rat Anti-Mouse CD8a | 561966 | BD Pharmingen | Rat |
| APC Hamster Anti-Mouse CD279 (PD-1) (J43) | 562671 | BD Pharmingen | Hamster |
| BV421 Rat Anti-Mouse IFN-γ | 563376 | BD Pharmingen | Rat |
| PE/Cyanine7 anti- mouse Granzyme B | 372214 | Biolegend | Rat |

**Table S2** **Quantitative PCR primers used in this study.**

| mRNA | Forward Primer (5’-3’) | Reverse Primer (5’-3’) |
| --- | --- | --- |
| CCL2 | TCACCTGCTGCTACTCATTCACC | TCTTTGGGACACCTGCTGCTG |
| GAPDH | GGTTGTCTCCTGCGACTTCA | TGGTCCAGGGTTTCTTACTCC |

**Table S3** **Gene ontology / Biological process (GO_BP) pathways.**

| GO ID | Description | RichFactor | pvalue | Gene ID |
| --- | --- | --- | --- | --- |
| 0050900 | leukocyte migration | 0.083 | 1.1565E-08 | Edn1/Ccl9/Spn/Slamf9/Csf1r/P2ry12/  C3ar1/Vcam1/Cxcl15/Ppbp/Ffar2/Cx3cl1/  Prtn3/Ptk2b/Dapk2/Spp1/Ccl20/Vav3/  Csf1/Itga1/Eps8/Plg/Ccl2/Il33/Spns2/  S1pr1/Wnt5a/Thbs1/Tnfrsf18/Itga7/  Dusp1/Serpine1/Gp2 |
| 0002697 | regulation of immune effector | 0.061 | 3.49084E-05 | Angpt1/Spn/C4bp/Clec2d/Cd28/Ffar2/  Siglecg/Apoa1/Spink5/Cfh/Serpinb9b/  Enpp3/Cd36/H2-T24/Masp1/Ccl2/  Raet1e/Il33/Serpinb9/Pglyrp1/Il18/  Wnt5a/Il18rap/Irf5/Dnajb9/Unc13d/  Sema7a/Gab2/Hmox1 |
| 0002274 | myeloid leukocyte activation | 0.082 | 2.20591E-06 | Mrgprb3/Mrgprb2/Ubd/Csf1r/Tff2/  Scnn1b/Cx3cl1/Tlr1/Fcgr4/Itgb6/Enpp3/  Tmem229b/Adgrf5/Il33/Il18/Wnt5a/  Il18rap/Thbs1/Unc13d/Gab2/Gpr137b/  Ndrg1/Hmox1 |
| 0030595 | leukocyte chemotaxis | 0.092 | 5.88407E-07 | Edn1/Ccl9/Slamf9/Csf1r/C3ar1/Cxcl15/  Ppbp/Ffar2/Cx3cl1/Ptk2b/Dapk2/Spp1/  Ccl20/Vav3/Csf1/Itga1/Ccl2/S1pr1/Wnt5a/Thbs1/Dusp1/Serpine1 |
| 0001818 | negative regulation of cytokine | 0.061 | 0.000871898 | Angpt1/Igf1/Serpinb1a/Cx3cl1/  Inpp5d/Apoa1/Gbp4/Trib2/Il33/  Muc16/Homer2/Pglyrp1/Tigit/Thbs1/  Klf2/Arg2/Ddit3/Cidea/Hmox1 |
| 0032640 | tumor necrosis factor production | 0.076 | 0.000114087 | Angpt1/Igf1/Spn/Slamf9/Csf1r/Cx3cl1/  Tlr1/Cd36/Nfatc4/Ccl2/Il33/Il18/  Wnt5a/Thbs1/Arg2/Arhgef2/Cidea |
| 0050777 | negative regulation of immune | 0.071 | 0.000260871 | Spn/Col3a1/C4bp/Clec2d/Inpp5d/Spink5/  Aurkb/Serpinb9b/Enpp3/Smpdl3b/Masp1/  Il33/Serpinb9/Pglyrp1/Arg2/Mmp12/Hmox1 |
| 0032680 | regulation of tumor necrosis | 0.074 | 0.000248752 | Angpt1/Igf1/Spn/Csf1r/Cx3cl1/Tlr1/  Cd36/Nfatc4/Ccl2/Il33/Il18/Wnt5a/  Thbs1/Arg2/Arhgef2/Cidea |
| 0061517 | macrophage proliferation | 0.333 | 0.000210996 | Csf1r/Cx3cl1/Csf1/Il33 |

**Table S4** **PPI node degrees.**

| Node | Identifier (10090) | Degree | Node | Identifier (10090) | Degree |  |
| --- | --- | --- | --- | --- | --- | --- |
| CCL2 | ENSMUSP00000000193 | 10 | IL18rap | ENSMUSP00000027237 | 1 |  |
| IL18 | ENSMUSP00000151002 | 9 | Spn | ENSMUSP00000049534 | 0 |  |
| Serpinb9 | ENSMUSP00000099002 | 7 | Clec2d | ENSMUSP00000032260 | 0 |  |
| Serpinb9b | ENSMUSP00000006392 | 7 | Dnajb9 | ENSMUSP00000015049 | 0 |  |
| CD36 | ENSMUSP00000133008 | 6 | Enpp3 | ENSMUSP00000020169 | 0 |  |
| Homx1 | ENSMUSP00000005548 | 5 | Gab2 | ENSMUSP00000004622 | 0 |  |
| IL33 | ENSMUSP00000025724 | 4 | H2-T24 | ENSMUSP00000109389 | 0 |  |
| Apoa1 | ENSMUSP00000034588 | 3 | Pglyrp1 | ENSMUSP00000032573 | 0 |  |
| CD28 | ENSMUSP00000027165 | 3 | Raet1e | ENSMUSP00000138022 | 0 |  |
| Cfh | ENSMUSP00000107607 | 3 | Sema7a | ENSMUSP00000042211 | 0 |  |
| Irf5 | ENSMUSP00000127021 | 2 | Siglecg | ENSMUSP00000005592 | 0 |  |
| Masp1 | ENSMUSP00000087327 | 2 | Spink5 | 1ENSMUSP00000066214 | 0 |  |
| Angpt1 | ENSMUSP00000022921 | 1 | Unc13d | ENSMUSP00000074549 | 0 |  |
| C4bp | ENSMUSP00000027657 | 1 | Wnt5a | ENSMUSP00000064878 | 0 |  |
| Ffar2 | ENSMUSP00000052600 | 1 |  |  |  |  |
